# Intermediate-Risk Pulmonary Embolism: Patients’ Stratification, Prognosis, and Therapeutic Options—Time to Pay Attention to the Middle Child

**DOI:** 10.3390/jcm14176215

**Published:** 2025-09-03

**Authors:** Sharon Shalom Natanzon, Mahmoud Mansour, Alexander Fardman, Fernando Chernomordik, Romana Herscovici, Shlomi Matetzky, Roy Beigel

**Affiliations:** 1 Intensive Cardiac Care Unit and Department of Cardiology, Division of Cardiology, Sheba Medical Center, Tel-Hashomer, Ramat Gan 52621, Israel; sharonnatanzon@gmail.com (S.S.N.); mahmoud.mansour.h89@gmail.com (M.M.); alexfardman@gmail.com (A.F.); fernandoandres.chernomordik@sheba.health.gov.il (F.C.); romana.herscovici@sheba.health.gov.il (R.H.); shlomi.matetzky@sheba.health.gov.il (S.M.); 2 Division of Cardiology, University of Florida College of Medicine, Jacksonville, FL 32209, USA; 3 The Gray Faculty of Medical and Health Sciences, Tel-Aviv University, Tel-Aviv 6997801, Israel

**Keywords:** pulmonary embolism, intermediate risk, imaging, stratification, therapy

## Abstract

Acute pulmonary embolism can range from being completely asymptomatic to causing life-threatening events, which underscores the importance of effective risk stratification. Intermediate-risk patients represent a distinct subgroup characterized by specific clinical, laboratory, and imaging features. Although the majority have favorable outcomes, a significant proportion may still experience adverse events, presenting an ongoing challenge in determining the optimal therapeutic approach. This comprehensive review explores the characteristics of intermediate-risk pulmonary embolism patients, focusing on key diagnostic and prognostic factors, current treatment practices, and the evolving role of novel, specifically catheter-directed interventions. We also provide an overview of current guideline recommendations and discuss recent advancements in the field.

## 1. Introduction

Intermediate-risk pulmonary embolism (PE) patients are a subgroup of PE patients with an elevated and significant risk for recurrent venous thromboembolism (VTE), hemodynamic compromise, and death [1,2] as compared to patients with low-risk PE.

This specific group of intermediate-risk patients is characterized by clinical, echocardiographic, radiologic, and laboratory variables. However, the optimal, and mainly the initial therapeutic approach for this group is still debatable [2,3,4,5] with some warranting a more aggressive approach than that currently recommended by the guidelines consisting of anticoagulation.

We focus on intermediate-risk PE patients reviewing current guidelines as well as the contemporary data which have accumulated in regard to different diagnostic and therapeutic options in these patients.

## 2. Definition and Epidemiology

VTE is a common disease with a prevalence of 100–200/100,000 in Europe as well as in the US [6]. About 60,000–100,000 Americans die each year from VTE and according to the Center for Disease Control and Prevention [7], 10–30% of them during the first month of diagnosis. VTE and especially PE symptoms vary and can make diagnosis challenging with sudden cardiac death being the first manifestation in up to 25% of patients [8].

The European Society of Cardiology (ESC) guidelines [1] divide PE patients into 3 distinctive sub-groups according to their mortality risk as part of the risk stratification. While high-risk PE patients are defined as those presenting with hemodynamic instability, on the other side of the spectrum are those at low risk who are hemodynamically stable without any findings suggesting cardiac, and particularly right ventricular (RV) involvement and without significant comorbidity. In between these two groups is the group of intermediate-risk PE patients which includes those who are hemodynamically stable, yet have positive findings suggesting RV involvement defined as either 1. Elevated cardiac biomarkers (i.e., troponin and/or brain natriuretic peptide) and/or 2. Evidence of RV dysfunction and/or enlargement either upon echocardiography or upon computed tomography (CT). Additionally, patients who do not have evidence of RV involvement but with significant comorbidities or clinical characteristics, which can be determined by the Pulmonary Emboli Severity Index (PESI) score of III-IV or a simplified PESI (sPESI) score of >0, are also classified as intermediate risk (further detailed) [1].

The hemodynamic response in PE depends on various factors including the thrombotic burden and magnitude of the embolus, the cardio-pulmonary reserve, and neuro-hormonal activation. The reduced pulmonary vascular bed increases RV afterload. Eventually, the right ventricle can dilate, become hypokinetic and fail at later stages. The high pressure in the right heart chambers can also cause deviation of the interventricular septum, causing an interventricular shift which further reduces left ventricular filling and consequently leads to further reduction in cardiac output [9]. All of these findings can be evident upon echocardiographic evaluation.

In the presence of RV dysfunction, there is a higher risk of complications and adverse events [9,10,11,12,13] as well as a higher mortality rate as opposed to those without RV involvement [13,14]. The physiology of the failing RV is related to both pulmonary vascular obstruction as well as release of vasoconstrictive agents causing increased afterload and RV strain [15].

According to various reports, the in-hospital mortality rate for intermediate-risk PE patients has been estimated to be in the range of 3–15% [16] with a 30-day mortality rate of 6–7.7% [2]. Intermediate-risk patients are further subdivided into 1. Intermediate–low-risk: patients with either elevated cardiac biomarkers or evidence of RV dysfunction upon imaging, or alternatively when both are negative but the patient has a high PESI/sPESI score (further detailed), or 2. Intermediate–high-risk when there is both evidence of elevated biomarkers as well as RV dysfunction upon imaging (either by echocardiography or CT).

While intermediate risk refers to the European classification of PE patients, the American College of Chest Physicians [17] categorizes the equivalent of intermediate-risk PE as submassive PE. Like their European counterparts, submassive PE patients are normotensive and hemodynamically stable with either evidence of RV involvement upon imaging (by echocardiography or CT) or evidence of elevated cardiac biomarkers. Unlike the ESC classification, there is no further subclassification of this group of patients. It is estimated that these patients account for 20–25% of the PE population with a mortality risk of up to 30% [18], which might relate to a higher risk population than those referred to by the ESC.

## 3. The Pulmonary Embolism Severity Index (PESI) Clinical Scoring System

As noted, one of the criteria for defining patients as intermediate risk relies on their clinical profile. One of the most validated and used scoring systems for risk stratification of patients with acute PE is the Pulmonary emboli severity index (PESI) or the simplified PESI (sPESI) score [19,20] (Supplementary Material Table S1). First described by Aujesky et al. [19], the PESI score has been shown to predict both short-term (30 and 90 days) as well as long-term mortality (up to 12 months) [21,22,23] in patients with acute PE (Table 1).

The PESI score has a practical function of identifying patients with low risk that may safely be treated at home without admission to the hospital [24,25]. Patients with low scores have a low risk of in-hospital mortality (PESI SCORE I–II 30 days’ mortality risk: 0–3.5% and sPESI-0: 0–2.1% 30 days’ mortality risk), whereas those in the higher risk group (PESI IV-V, sPESI > 1) have an up to 18% in-hospital mortality risk. The PESI score is highly validated with high sensitivity (91%) and negative predictive value (99%) for overall mortality [24]. Wicki et al. [26] found the PESI score to be superior to the older GENEVA score in identification of patients with an adverse 30-day outcome.

A low PESI score can offer the clinician the opportunity to avoid echocardiography and laboratory assessment [1]. However, caution is warranted, as there are still patients with a low PESI/sPESI score which might be misclassified if evaluated solely by the PESI score [27]. Cordeanu et al. [28] revealed that one-third of patients with sPESI-0 had at least one element justifying their assignment to an intermediate-risk category. While the PESI/sPESI score has been shown to predict which PE patients are at low risk of complications [29], its ability to identify patients prone to deterioration has been questioned by some [27,30] and as such it is recommended only for initial risk stratification and classification of patients as either low or intermediate risk. Different trials indicate the need for using a combination of clinical variables, imaging data, and laboratory data upon deciding on the best therapeutic management in intermediate-risk PE patients [31]. Currently there is a growing need for prospective trials evaluating specifically intermediate-risk PE patients and identifying predictors for clinical deterioration and/or the need for escalation therapy in this patient population [32].

## 4. Lower Extremity Doppler

In patients diagnosed with PE, lower extremity Doppler ultrasound plays a crucial role in identifying concurrent deep vein thrombosis (DVT), which is present in approximately 50–70% of cases and serves as the primary source of emboli, thereby aiding in risk stratification for recurrent thromboembolism and guiding anticoagulation duration [1,17]. Special circumstances warranting additional therapeutics beyond standard anticoagulation include high-risk PE with hemodynamic instability or RV dysfunction, as well as concerning thrombus features such as a free-floating thrombus in the lower extremity veins or thrombus in transit within the heart (e.g., right heart chambers or across a patent foramen ovale) [14,33,34]. These findings, which might be the sole findings without any additional high-risk features, can be associated with increased embolic potential and high mortality rates and might influence decisions toward additional therapies. For instance, a floating thrombus may prompt consideration of inferior vena cava filters or escalated interventions in select cases, while a thrombus in transit might justify mechanical thrombectomy (e.g., catheter-directed or surgical) to rapidly reduce clot burden and prevent catastrophic embolization, even in hemodynamically stable patients. The evidence regarding the benefit of such therapies is not solid and treatment should be individualized based on patients’ bleeding risk and overall clinical status.

## 5. Non-Invasive Evaluation and Stratification of PE Patients

### 5.1. Echocardiography

**Figure 1:**
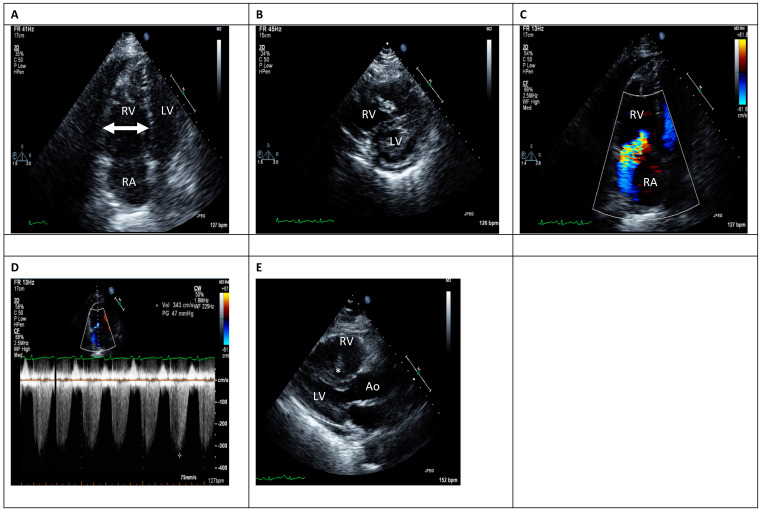
Echocardiographic findings suggestive of RV involvement in intermediate-risk PE. (**A**) Apical 4-chamber view showing right ventricular dilation. (**B**) Short-axis view showing interventricular septum deviation as well as a visible right-sided thrombus (asterisk). (**C**) Severe tricuspid regurgitation. (**D**) Pulmonary artery pressure Doppler curve demonstrating an increased pulmonary artery pressure. (**E**) Parasternal long-axis view showing a visible right-sided thrombus (asterisk). Ao—Aorta; LV—left ventricle; RA—Right atrium; RV—right ventricle.

Echocardiography plays an essential role in the risk stratification of patients presenting with PE. The main echocardiographic findings suggesting RV involvement in patients presenting with PE are shown in Figure 1 and summarized in Table 2. When assessing the RV and the degree of its involvement in PE patients, several points are of particular interest and should be emphasized:

1. A normal RV function does not rule out the presence of a PE. The negative predictive value of echocardiography for PE is about 40–50% [1].
2. As the RV morphology is complex due to its crescent shape, the definition of RV dysfunction can be somewhat vague [35,36]. Main echocardiographic findings suggestive of RV dysfunction include the following: 1. RV dilatation and systolic dysfunction, often with preserved contractility of the RV apex, a pattern known as McConnell’s sign, which has a specificity of 94% [37]. It should be kept in mind that the crescent shape of the RV may lead to high inter-observer variation in assessment of RV function [38]. 2. Signs suggestive of RV pressure overload such as shift of the interventricular septum to the left, which can be seen as a D-shape in the short-axis view (Figure 1B).

**Table 2:** Echocardiographic findings suggestive of RV involvement in intermediate-risk PE.

Echocardiographic Finding	Description	Comments
McConnell’s sign	Normal excursion of the right ventricular apex with hypokinesis of the mid-free wall segment [37]	There is a poor PPV (45–67%) for acute PE diagnosis [39]. An additional study found the McConnell’s sign to be correlated with a high thrombotic burden in particular central or multi-lobar PE, which may be associated with a higher risk of clinical deterioration. This association is still debatable [40].
RV dysfunction	No standard definition	Shown to be a marker of adverse events [41,42]. There is a high inter-observer variability.
RV dilatation Figure 1A	End diastolic diameter > 30 mm, RV/LV end diastolic diameter > 0.9 [43]	30% of patients with normotensive pulmonary embolism are diagnosed with RV dilation [11]—an independent risk of in-hospital mortality. High inter-observer variability in measurements.
Interventricular septum shift Figure 1B	“D”-shaped septum in the short-axis view is a secondary sign for increased RV pressures	Leftward interventricular septal shift may be associated with increased mortality
Tricuspid annular pane systolic excursion (TAPSE)	TAPSE < 1.6 cm	Shown to predict both 30-day and all-cause mortality as well as PE-specific mortality [44,45]. Main limitation—low inter-observer variability
Systolic pulmonary artery pressure (SPAP) and mean PAP (MPAP) estimation Figure 1D	Measured using the simplified Bernoulli equation: PAP = 4V^2^ + right atrial pressure (RAP)	Elevated PAPs are associated with a higher risk of both in-hospital and ICU mortality [46]. Easily obtained when there is an adequate tricuspid regurgitation signal. (Figure 1C). Evaluation of RAP via IVC parameters [47] can be problematic and a main cause for over/under estimation of PAPs Elevated PAP is not part of risk stratification among PE patients according ESC guidelines [1]
Visible Right Sided thrombi or thrombus in transit Figure 1E	An infrequent finding	A large registry found it to be associated with more complicated presenting symptoms (tachycardia, lower blood pressure) as well as higher short-term mortality among PE patients [33]

### 5.2. Additional Echocardiographic Parameters

#### 5.2.1. Tricuspid Annular Plane Systolic Excursion (TAPSE)

TAPSE is a rather simple quantitative method that reflects RV function [44], which has been shown to be a prognostic factor in various clinical scenarios including patients with pulmonary hypertension. Lobo et al. [45] have found a TAPSE < 16 mm to be a predictor of both 30-day all-cause mortality as well as PE-specific mortality. Pruszczyk et al. [11] examined the prognostic value of echocardiographic parameters among 411 normotensive PE patients. The clinical endpoint included 30-day PE-related death or the need for thrombolysis. Multi-variable analysis found TAPSE to be the only prognostic parameter (HR: 0.64, 95% CI: 0.54 to 0.7; *p* < 0.0001). Furthermore, a higher TAPSE (>20 mm) can be used to identify a group of patients with a low risk for adverse outcomes. TAPSE, in addition to its ability to evaluate RV function as well as a validated prognostic factor, has been shown to have a low inter-observer variability [48], which makes it more accurate and less operator-dependent. In an analysis by Khemasuwan et al., TAPSE correlated with the need for mechanical ventilation and survival [46]. While TAPSE is somewhat established as a prognostic factor among PE patients, it should be remembered that patients presenting with PE can still have normal values. Thus, TAPSE should serve as one of many parameters for a comprehensive assessment and prognostication.

#### 5.2.2. Pulmonary Artery Pressure (PAP)

While an elevated systolic PAP has low specificity for diagnosis of PE and cannot be used by itself in the initial diagnostic process, an elevated systolic PAP has been shown to be a prognostic factor for adverse outcomes in PE patients. Khemasuwan et al. [46] have shown elevated RV pressure to be associated with a higher risk of both in-hospital and intensive cardiac unit mortality.

##### Right Ventricular–Pulmonary Artery Coupling

A low TAPSE/PASP ratio was associated with increased mortality in acute PE patients and can be useful for further risk stratification in intermediate-risk patients [49].

#### 5.2.3. Echocardiographic Response to Therapy

After initiation of therapy (either anticoagulation or fibrinolysis), echocardiographic signs of RV dysfunction can show resolution as early as within several hours. Among 101 PE patients that were hemodynamically stable but with evidence of RV dysfunction upon echocardiography [50] within 24 h of therapy, RV wall motion improved in 39% of patients that received fibrinolysis as opposed to in 17% of those that received heparin. RV wall motion worsened in 2% and 17% of the patients, respectively (*p* = 0.005). Kline et al. [51] enrolled 200 PE patients classified as submassive and treated with heparin, of whom 21 (10%) required escalation therapy with alteplase due to circulatory shock or respiratory failure. The percentage of patients with RV hypokinesis decreased from 20% to 7% in patients treated with anticoagulation as opposed to from 57% to 6% in those treated with fibrinolysis. Nearly half of the patients that were treated with anticoagulation had persistent elevated RV systolic pressure and pulmonary hypertension and 46% of the patients had dyspnea either at rest or with evidence of exercise intolerance. None of the patients treated with thrombolysis had persistent elevated RV systolic pressure and 28% reported dyspnea either at rest or with exercise. However, in a subgroup analysis of the largest trial to date, the PEITHO trial, which prospectively randomized 709 acute intermediate–high-risk PE patients comparing treatment with TPA versus heparin, there were no long-term differences in mortality or residual RV dysfunction/pulmonary hypertension between the two groups [52].

### 5.3. Computed Tomography (CT) (Figure 2)

CT is the gold standard imaging modality for the diagnosis of PE [1], and stratification of PE patients into risk groups can also be performed based upon CT findings. Most of these findings relate to the RV anatomy while the prognostic significance of the extent of PE (clot burden in the vascular bed) as well as the anatomic thrombi location remains debatable [53,54].

**Figure 2:**
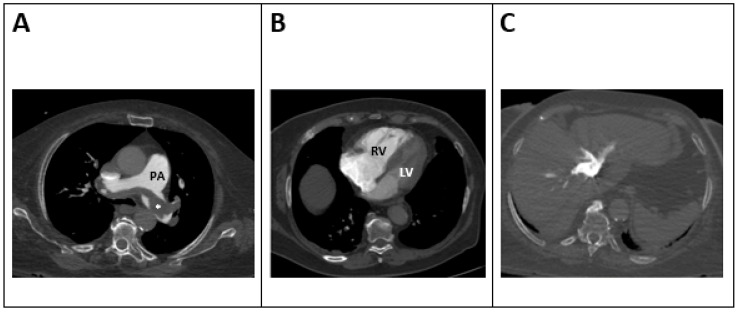
Common finding upon computed tomography in patients with intermediate-risk/submassive PE. (**A**) The pulmonary artery is shown with a filling defect consistent with a saddle embolus (asterisk). (**B**) RV dilation in 4-chamber view with an RV/LV diameter ratio > 1. (**C**) White contrast media is shown within the hepatic veins, demonstrating IVC reflux of contrast to hepatic veins. LV—left ventricle; PA—pulmonary artery; RV—right ventricle. (**A**) Computed Tomography Angiography demonstrating saddle pulmonary embolus; (**B**) Computed Tomography Angiography demonstrating right ventricle dilation in 4 chamber views, which looks equal or larger than LV; (**C**) Computed Tomography Angiography demonstrating inferior vena cava reflux which suggests high right-sided filling pressures.

#### 5.3.1. RV/LV Ratio

The RV/LV ratio upon CT is the parameter with the highest correlation to prognosis [55]. The RV/LV ratio can be measured either in the 4-chamber view or the axial view. A ratio of >0.9 is considered abnormal and is associated with higher in-hospital mortality as well as PE-related mortality [55]. Mansencal et al. [56] found that an RV/LV ratio > 1 has a high sensitivity (88%) and specificity (88%) for the diagnosis of PE as well as for identifying high-risk patients with RV dysfunction.

#### 5.3.2. Evaluation of Thrombotic Clot Burden

Two CT scores have attempted to establish the role of “clot burden” as part of the risk stratification among PE patients: The Mastora severity index provides a detailed description of the anatomical location of the clot as well as percentage of luminal obstruction, while the Qanadli score is based on quantification of the obstructed pulmonary vessels. Conflicting results have emerged regarding the prognostic role of clot burden as well as its correlation with RV dysfunction [57,58,59,60,61,62,63]. Furthermore, both the Mastora and Qanadly scores do not include the pulmonary vessels located distal to sub-segmental arteries which may contribute significantly to clot burden. Due to their complexity and equivocal trial results, there is no routine clinical use of these scores among PE patients.

Additional CT findings which might be associated with high-risk patients, which include IVC contrast reflux [64], decreased left atrial volume [65], as well as right atrial/RV ratio [66], are detailed in Table 3.

Currently, no scoring system takes into account both clot burden or other CT parameters in addition to patients’ comorbidities and presents findings which have a critical influence on prognosis. Hence, an allegedly small clot burden upon CT in those without cardiopulmonary reserve may lead to hemodynamic compromise. As was concluded in past trials, no single CT finding can currently guide therapeutic decisions in PE patients.

### 5.4. Ventilation-Perfusion (V/Q) Scan

Ventilation-perfusion (V/Q) scanning plays a pivotal role in the diagnosis of PE, serving as a non-invasive nuclear medicine technique that evaluates mismatches between lung ventilation and perfusion to identify vascular obstructions, particularly when CT is contraindicated due to factors like renal insufficiency, severe contrast allergy, or pregnancy, where V/Q might offer, in some cases, lower radiation exposure to the breasts and fetus [69,70,71]. Guidelines from the European Association of Nuclear Medicine (EANM) strongly recommend V/Q single-photon emission computed tomography (SPECT) for its high diagnostic accuracy, with sensitivity ranging from 96 to 99% and specificity of 96–98%, enabling reliable PE detection even in patients with comorbidities such as chronic obstructive pulmonary disease (COPD), heart failure, or pneumonia, and reducing non-diagnostic rates to 1–4% [69]. A systematic review and meta-analysis further support this, reporting pooled sensitivity up to 0.98 (95% CI: 0.95–0.99) when interpreting high, non-diagnostic, and low-probability scans as positive, alongside specificity of 0.36 (95% CI: 0.27–0.45), highlighting V/Q’s strength in safely ruling out PE with comparable false-negative rates to CT at 3-month follow-up [72]. Interpretation relies on established criteria such as the modified PIOPED II (Prospective Investigation of Pulmonary Embolism Diagnosis II), in which high-probability scans (≥2 large mismatched segmental defects) indicate the presence of PE. Advancements in SPECT/CT hybrid imaging further provide anatomic correlation, improving specificity and addressing limitations of planar techniques, such as the higher nondiagnostic rates reported in earlier studies [69,71]. While V/Q scans aid in the diagnosis of PE, they do not provide additional information regarding RV function. Risk stratification in patients with confirmed PE can be further refined using echocardiography and cardiac biomarkers (as further discussed).

## 6. Laboratory Findings

As part of the comprehensive risk stratification of PE patients, in addition to clinical and imaging findings, both the ESC [1] and the ACCP guidelines [17] support cardiac biomarkers to classify the risk of adverse outcomes. Among these, the most validated biomarkers are troponin (Table 4A) and BNP/NT-proBNP (Table 4B). Additional biomarkers are currently being evaluated for their utility in risk-stratifying patients with PE (Table 4B).

Elevated values of troponin usually signify myocardial injury. In patients with PE, elevated troponin levels can be secondary to either RV strain and/or myocardial micro-infarction. Of note, as the RV mass is much smaller than that of the left ventricle, peak troponin levels in PE patients which originate due to RV involvement are generally lower than those measured during an acute coronary syndrome involving the left ventricle [97].

Due to its high sensitivity and negative predictive value, a negative troponin test identifies patients at lower risk for adverse outcomes. Some studies have challenged the role of troponin as a prognostic factor in normotensive PE patients [98], especially in those with a very low PESI or sPESI score [2]. Nevertheless, both the ESC [1] as well as the ACCP guidelines [17] define the presence of elevated troponin as a risk for adverse outcomes, classifying these patients as non-low-risk.

In a large meta-analysis of 38 trials [73] evaluating normotensive PE patients, elevated troponin levels were associated with a higher overall mortality as well as PE-related mortality. In addition, elevated troponin levels were associated with adverse events defined as a composite of death, need for thrombolysis, endotracheal intubation, catecholamine infusion for sustained hypotension, cardiopulmonary resuscitation, or recurrent PE. There is concrete evidence to correlate positive troponin and RV dysfunction as well as a complicated in-hospital course [99] (Table 4A). Brain natriuretic peptide (BNP) and NT-proBNP are also markers of RV dysfunction. BNP is released as a response to myocyte stretch [100]. The ESC guidelines as well as ACCP guidelines use BNP as a marker for adverse events in PE patients. A large meta-analysis [82] found significant correlation between high BNP levels and clinical deterioration in PE patients as well as RV dysfunction. In addition, BNP was found to have a high negative predictive value [100] such that normal values are associated with a more benign clinical course.

Heart-type fatty acid-binding protein (H-FABP), a cytoplasmic protein which leaks to the cytoplasm during myocyte ischemia, has recently emerged as a prognostic factor among PE patients [88,90]. It is a nonspecific marker that was tested in PE patients as well as in the setting of an ACS [101]. The advantage of H-FABP is its pharmacokinetics. In contrast to BNP and troponin, which can take time to reach elevated levels following the initial cardiac insult [73], H-FABP plasma concentration rises rapidly and can be measured within 90 min [87]. Several studies have found H-FABP to be a good predictor of mortality [87,88,91] as well as for clinical deterioration [88] and the presence of RV dysfunction [89,91] (Table 4B).

D-Dimer is a protein, a product of fibrin degradation, which can be seen after fibrinolysis of a blood clot. As D-dimer can be elevated in a variety of clinical scenarios, it is not specific for the diagnosis of VTE. However, due to its a high negative predictive value, its use in clinical practice is to exclude VTE in those deemed to be at low to intermediate risk for VTE [102].

A meta-analysis of 22 studies with both stable and unstable PE patients by Becattini et al. [94] evaluated the prognostic role of D-Dimer. Elevated D-dimer was associated with increased short-term and 3-month mortality. However, different trials used different prognostic cut-off levels as well as different assays, so an absolute prognostic value could not be determined. Several studies evaluated the correlation between D-Dimer and RV dysfunction on CT angiography, but with conflicting results [103,104,105,106].

Plasma lactate is a useful non-specific but sensitive marker of tissue hypoperfusion and may be elevated before overt hypotension develops. Several studies [92,93] have tested the ability of lactate to monitor hemodynamically stable PE patients. Vanni et al. [93] found lactate to correlate with PE-related mortality as well as with hemodynamic collapse, defined as the need for cardiopulmonary resuscitation, a systolic blood pressure < 90 mm Hg for at least 15 min, the need for catecholamine administration, or the need for mechanical ventilation.

While cardiac biomarkers such as high-sensitivity troponin and NT-proBNP reflect myocardial injury and wall stress [86,107], vasopressin and its more stable surrogate, copeptin, may represent a novel pathophysiological axis in assessing PE severity. In acute PE, the abrupt rise in RV afterload can reduce left ventricular preload, leading to decreased cardiac output and potential shock. As vasopressin and copeptin are released in response to physiological stress and hypotension [95,108], their elevation may reflect systemic responses to hemodynamic compromise secondary to RV failure.

A prospective single-center study of 268 normotensive PE patients [95] demonstrated that copeptin levels ≥24 pmol/L were associated with a 5.4-fold increased risk of adverse 30-day outcomes. This risk was further amplified when copeptin was integrated with troponin and NT-proBNP in a stepwise biomarker-based risk stratification approach. Similarly, a European multicenter study involving 843 normotensive acute PE patients [96] found that copeptin levels ≥ 24 pmol/L were linked to a 6.3-fold increased risk of adverse outcomes and a 7.6-fold increased risk of PE-related death. These findings support the role of copeptin as a marker of hemodynamic deterioration due to acute RV failure, with potential implications for more intensive therapeutic strategies.

Notably, the prognostic utility of copeptin remained independent in multivariable models that accounted for other established predictors of adverse outcomes and PE-related mortality.

## 7. Treatment of Patients with Intermediate-Risk/Submassive PE

### 7.1. Medical Therapy

**Figure 3:**
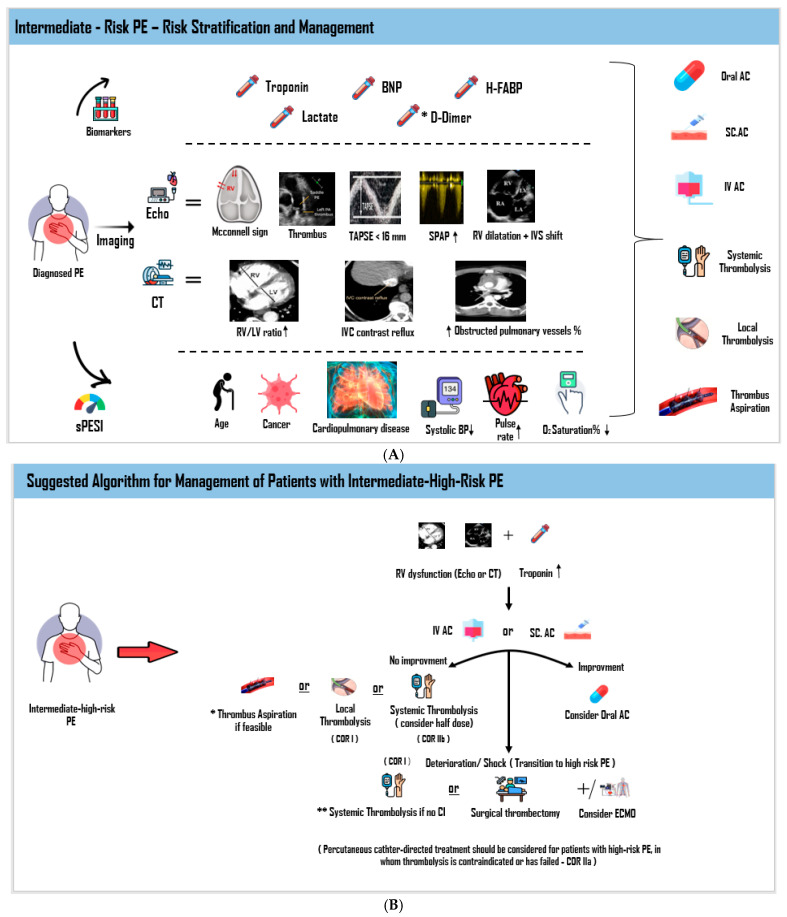
(**A**) PE Risk Stratification and Management. Risk stratification and management of intermediate-risk pulmonary embolism (PE). Assessment integrates biomarkers, imaging findings (echocardiography and CT), and the sPESI score. Treatment ranges from anticoagulation to escalation therapies, including systemic thrombolysis, catheter-directed interventions, or thrombus aspiration in selected patients. (**B**) Intermediate–high-risk PE management. Patients with intermediate–high-risk PE are typically treated initially with either intravenous or subcutaneous anticoagulation. If there is no clinical improvement, treatment may escalate to thrombus aspiration, local catheter-directed thrombolysis, or reduced-dose systemic thrombolysis. Should the condition deteriorate to high-risk PE, the treatment of choice becomes full-dose systemic thrombolysis or surgical thrombectomy with ECMO being an option for maintaining immediate hemodynamic stability [109]. AC = anticoagulation; CI = contraindication; COR = Class of recommendation; ECMO = extra corporeal membrane oxygenation; PE = pulmonary embolism. * Depending on the size, location, and age of the thrombus. ** Absolute CI for systemic thrombolysis: 1. History of hemorrhagic stroke or stroke of unknown origin. 2. Ischemic stroke in the previous 6 months. 3. Central nervous system neoplasm. 4. Major trauma, surgery, or head injury in previous 3 weeks. 5. Bleeding diathesis. 6. Active bleeding. The arrow means increased RV/LV ration.

Perhaps the most controversial issue regarding patients with intermediate-risk PE is the acute management. On the one hand, these patients are at a non-negligible risk for developing adverse events as well as the need for more aggressive therapy, while on the other hand, the risk might not mandate routine administration of aggressive therapy (i.e., thrombolysis), which can put the patients at risk of adverse events. The hallmark of therapy is presented in Figure 3A.

### 7.2. Anticoagulation Therapy

#### 7.2.1. Parenteral Anticoagulants

Anticoagulation is the mainstay of therapy for intermediate-risk PE patients. Anticoagulation therapy in patients with PE is divided into several phases: 1. acute (initial 5–10 days), 2. Long term (10 days–6 months), and 3. Extended (beyond 6 months). For intermediate-risk patients, acute phase treatment includes initiation of either unfractionated heparin (UFH), LMWH, bivalirudin, or fondaparinux. Mainly, the preferred parenteral anticoagulation regimen typically involves the use of LMWH or fondaparinux. These options are favored due to their lower risk of inducing major bleeding and heparin-induced thrombocytopenia compared to UFH. However, in cases when thrombolysis is still under consideration (mostly in intermediate–high-risk PE cases), either UFH or bivalirudin might be considered as the preferred agent of choice due to their shorter half-life and better monitoring of effect prior to, during, and after administration of thrombolysis. UFH might also be preferred in very obese patients and in those with severe renal impairment (creatinine clearance < 30 mL/min) [1].

#### 7.2.2. Oral Anticoagulants

Until about a decade ago, vitamin K antagonists (VKAs), mainly warfarin, were the mainstay of oral anticoagulation therapy for many years. Warfarin is usually initiated along with parenteral anticoagulants along with monitoring INR (therapeutic range of 2–3). However, in recent years, the use of non-VKA oral anticoagulants (NOACs) has emerged as an alternative to warfarin. All four available NOACs have been evaluated in acute PE patients. It should be noted that patients with intermediate-risk PE were not excluded in the trials evaluating the efficacy and safety of NOACs.

The RE-COVER and RECOVER II trials [110,111] compared dabigatran to warfarin for the treatment of VTE (both PE and DVT). Both trials included a total of 5128 patients; among them, approximately 20% had PE and 9% had both DVT and PE. Hemodynamically unstable patients were excluded from these two trials. Dabigatran was found to be non-inferior to warfarin in the 6-month incidence of recurrent symptomatic or fatal VTE. There is no sub-analysis data regarding dabigatran treatment in the intermediate-risk/submassive PE groups.

EINSTEIN-PE [112] evaluated the use of Rivaroxaban in acute PE patients compared to warfarin. A total of 4833 patients underwent randomization. Patients planned for reperfusion therapy either by thrombolysis or pulmonary embolectomy were excluded. Rivaroxaban was shown to be as good as warfarin for treating VTE, with similar bleeding rates. While in this study a significant portion of patients had extensive disease per CT (25% of patients had multiple lobes involvement; 12% of the patients were admitted to an intensive care unit), there is an absence of data regarding the presence of RV dysfunction or dilatation upon imaging studies and whether or not patients had elevated cardiac biomarkers.

The AMPLIFY study [113] evaluated treatment with Apixaban compared with warfarin or enoxaparin in VTE patients. The study included 5395 patients with an acute VTE event; of these, approximately 50% had PE. The primary efficacy outcome was recurrent symptomatic VTE or death related to VTE. Apixaban was shown to be non-inferior to conventional treatment with warfarin in the primary efficacy outcome. In addition, Apixaban was associated with significantly less bleeding (both major bleeding and the combined outcome of major and clinically relevant minor bleeding). Again, there is a lack of specific data regarding categorization of patients to subgroups such as intermediate-risk/submassive PE.

The Hokusai-VTE trial [114] evaluated Edoxaban therapy for an acute VTE event in comparison to enoxaparin/warfarin. The trial included 4921 patients with DVT and 3319 patients with PE. Edoxaban was shown to be non-inferior to warfarin in regards to efficacy in treating VTE and have less episodes of fatal and intra-cranial bleeding. In contrast to previous NOAC trials, the Hukosai-VTE had a subgroup analysis of patients with RV dysfunction as well as elevated cardiac biomarkers. Approximately one-third of patients included in this study had RV dysfunction and elevated BNP levels, and in this subgroup of patients, Edoxaban was shown to be as good as warfarin.

The minimal duration of treatment with anticoagulation for patients with PE is 3 months [1], with the risk of recurrent PE being 2.5%/year in those who are regarded in the past as “provoked” PE and 4.5%/year in “unprovoked” PE [115]. Predicting the recurrence risk, especially in those who were once considered as “unprovoked” PE, is complex and challenging and thus affects the duration of treatment. Currently there are no specific recommendations for long-term therapy based upon the extent of the disease. A recent study from the PADIS-PE [116] database suggested that among patients with a first episode of unprovoked PE, age, pulmonary vascular obstruction index (measured either at diagnosis or at 6 months of anticoagulation), and the presence of anti-phospholipid antibodies were found to be independent predictors of recurrent VTE; however, this is yet to be largely validated.

#### 7.2.3. Thrombolysis

The aim of thrombolytic therapy is to rapidly restore pulmonary vascular patency and reverse RV strain and dysfunction within hours [50]. While in high-risk patients, several trials have proved thrombolysis to be associated with lower PE-related mortality and with reduction in recurrent VTE and death [117,118,119], intermediate-risk PE patients are more complex. Due to a relatively lower mortality risk, it is extremely difficult to prove survival benefit among intermediate-risk patients; therefore, many trials have used the concept of combined outcomes [120].

Thrombolysis is associated with an increased risk of bleeding complications in up to 30% of patients [121], with an estimated major bleeding rate of 6–7% [121,122], as well as an increased risk for intra-cranial hemorrhage in 2% [122]. The risk of bleeding is assumed to be significantly higher among elderly patients. In the PEITHO trial [122], age > 75 was associated with significantly more extra-cranial bleeding when given thrombolysis compared to placebo (OR 20.38, CI 2.69–154.5, *p* = 0.09 for interaction). Mikkola et al. [123] described a fourfold higher risk of bleeding when thrombolysis was given to patients > 70 years old, compared to patients < 50 years old with a 4% increased risk of bleeding for each incremental year of age.

There have been a few studies evaluating the use of fibrinolysis as compared to anticoagulation therapy in normotensive PE patients. While some trials used the definition of “Submassive PE”, others followed the ESC guidelines’ definition and presented data on “Intermediate-risk” PE patients. A complete comparison of all trials performed is detailed in Table 5. Initial results of small randomized trials [120,124,125] as well as meta-analysis [126] suggested that patients with submassive or intermediate-risk PE might benefit from thrombolytic therapy. However, in the randomized PEITHO trial, where 5.0% of initially anticoagulated patients suffered either hemodynamic deterioration and/or death, thrombolytic therapy in intermediate–high-risk PE patients reduced the risk of hemodynamic collapse, but was counterbalanced by a higher risk of bleeding (major bleeding 11.5% vs. 2.4%, *p* < 0.001), and an overall negative net clinical benefit [122]. Moreover, during a long-term follow-up of 37.8 months, thrombolysis did not have any effect with regard to mortality or on reduction in residual dyspnea or RV dysfunction [52]. Following the PEITHO results, the current ESC guidelines [1] do not support the routine use of thrombolysis in intermediate–high-risk patients. Rather, the guidelines currently endorse an approach consisting of close monitoring, and once hemodynamic deterioration is noted, rescue thrombolysis should be administered. While the ACCP guidelines do not support thrombolysis for non-hypotensive PE patients, they do give a recommendation for thrombolysis in patients who deteriorate after initiation of anticoagulation and who have an acceptable bleeding risk. The ACCP guidelines also highlight the need for close monitoring in those who present with severe symptoms and limited cardio-pulmonary reserve. This recommendation was reinforced in the second update of the CHEST guideline and expert panel report [127].

Several trials explored the idea of lower-dose fibrinolytic therapy. The MOPEET trial [128] evaluated 121 patients with “Moderate PE” (defined as the presence of a thrombus in >two lobar arteries or left/right pulmonary artery. RV dysfunction or biomarker elevation were not mandatory inclusion criteria) that were randomized to low, “safe dose”, fibrinolytic therapy (<50% of standard dose) or placebo. The primary outcome of pulmonary hypertension and the composite end point of pulmonary hypertension and recurrent PE at 28 months were significantly lower in the low-dose fibrinolysis group (16% vs. 57%, *p* < 0.001; 16% vs. 63%, *p* < 0.001, respectively). No bleeding events occurred in either group and no significant difference was observed in mortality or recurrent PE. In a meta-analysis by Zhang et al. [148], reduced-dose thrombolysis as compared to full-dose thrombolysis was shown to have similar efficacy but to be safer with fewer bleeding events. In addition, compared with heparin, low-dose rtPA did not increase the risk of major bleeding for eligible PE patients. These results raise the hypothesis of administering low-dose fibrinolysis that may be both effective and safe. Currently, the PEITHO-3 trial [147], evaluating the administration of reduced-dose thrombolysis vs. standard therapy with anticoagulation for intermediate–high-risk PE patients, is ongoing. The study’s results will be available soon and might provide us with more therapeutic options for intermediate–high-risk PE patients.

#### 7.2.4. Percutaneous Catheter-Directed Interventions (CDIs)

CDIs have the advantage of providing several approaches [149]; among them are aspiration of large emboli as well as injecting local thrombolysis directly to the obstructed vessel and clot, thus facilitating lysis with potential reduction in bleeding complications. Currently, according to the ESC guidelines, percutaneous intervention should be reserved for high-risk patients where thrombolysis has failed or is contraindicated (class IIa level of evidence C recommendation) [1]. A recent consensus statement by the ESC working group of pulmonary circulation and RV function and the European association of percutaneous cardiovascular interventions suggests that these interventions can be used prior to the use of systemic thrombolysis, even in the absence of contraindications, for intermediate–high-risk PE patients who have failed therapy with anticoagulation [150].

There are currently several major devices used for CDIs that could be divided into two groups: pharmacomechanical catheter-assisted-directed thrombolysis (CDT) and catheter-directed aspiration. Pharmacomechanical CDT is performed using ultrasound-assisted catheter-directed thrombolysis (CDT) for clot separation (EKOS Corporation, Bothell, WA, USA). Catheter-directed aspiration is performed either manually using a guide catheter or Pronto XL catheter, or via dedicated devices such as large-bore aspiration thrombectomy with the Inari FlowTriever system (Inari Medical Inc., Irvine, CA, USA), aspiration thrombectomy with the Indigo aspiration system, penumbra, (Alameda, CA, USA), or suction with the Angiovac cannula (Angiodynamics, Inc., Latham, NY, USA).

While most of the data comparing CDI with conventional anticoagulation in intermediate–high-risk PE patients derives from observational or retrospective trials, currently the most evidence has been accumulated for 1. Catheter-directed thrombolysis with the EKOS system and 2. Catheter-directed aspiration with the FlowTriever system and to a lesser extent the Indigo aspiration system.

The main findings from trials evaluating the various CDI methods are summarized in Table 5. Studies evaluating the EKOS system include the ULTIMA trial (Ultrasound Accelerated Thrombolysis of Pulmonary Embolism) [131], which was an open-label randomized clinical trial demonstrating the superiority of ultrasound-assisted thrombolysis in reversing RV dilation at 24 h. The device proved to be safe without additional major bleeding events compared to standard anticoagulation. The CANARY trial (Catheter-Directed Thrombolysis Versus Anti Coagulation Monotherapy in Patients with Acute Intermediate–High-Risk Pulmonary Embolism) [132], which was prematurely stopped due to COVID-19, did demonstrate a beneficial effect in reversing RV dilation at 3 months of follow-up. Two additional prospective single-arm studies were the Prospective, Single-Arm, Multicenter Trial of Ultrasound-Facilitated, Catheter-Directed, Low-Dose Fibrinolysis for Acute Massive and Submassive Pulmonary Embolism: The SEATTLE II Study [134] and Pulmonary Embolism Response to Fragmentation, Embolectomy, and Catheter Thrombolysis (PERFECT) [151] trials. Both demonstrated a decrease in PAP. The main limitations of these studies were the non-comparator group as well as lack of hard outcomes and a small sample size. Currently, the Higher-Risk Pulmonary Embolism Thrombolysis (HI-PEITHO) study [142] is an ongoing multinational, multicenter randomized controlled comparison trial which will randomize 1:1 intermediate–high-risk PE patient to treatment with ultrasound-facilitated catheter-directed thrombolysis plus anticoagulation, versus anti coagulation alone. The results of the HI-PEITHO trial are expected to be published within the upcoming year and will definitely have an impact on the recommendations for treatment of patients with intermediate–high-risk PE.

Major studies evaluating aspiration thrombectomy to date include the Prospective, Single-Arm, Multicenter Trial of Catheter-Directed Mechanical Thrombectomy for Intermediate-Risk Acute Pulmonary Embolism (FLARE) study [138], which evaluated the Inari FlowTriever Retrieval system in patients with intermediate–high-risk PE. Inclusion criteria included hemodynamically stable patients without vasopressors, heart rate < 130 BPM, systolic blood pressure > 90 mmhg, and an RV/LV ratio ≥ 0.9. Although this was a single-arm study, the FlowTriever System was found to be safe and effective with significant improvement in RV/LV ratio. The recently published Large-Bore Mechanical Thrombectomy Versus Catheter-Directed Thrombolysis in the Management of Intermediate-Risk Pulmonary Embolism (PEERLESS) trial [140] was a prospective, open-label trial evaluating the efficacy and safety of large-bore mechanical thrombectomy using the Inari FlowTriever Retrieval system versus several CDT methods among intermediate–high-risk PE patients. There was no difference between the groups in regards to 30-day mortality. The large-bore mechanical thrombectomy group had significantly lower rates of clinical deterioration and/or escalation to bailout therapy and less post-procedural intensive care unit days compared to CDT.

The Indigo Aspiration System for Treatment of Pulmonary Embolism (EXTRACT-PE) [141] trial was a prospective, single-arm trial evaluating the efficacy and safety of the Indigo aspiration system. A total of 119 patients were enrolled at 22 U.S. sites. There was significant reduction in the RV/LV ratio and a low major adverse events rate.

A summary of a suggested treatment algorithms for patients with intermediate–high-risk PE is shown in Figure 3B.

While the results of important ongoing trials in the field of CDI are still much anticipated, as detailed in Table 5, much has been learned to date which can aid and already help physicians integrate these techniques into different clinical scenarios. For instance, aspiration thrombectomy might be best utilized in cases of proximal or saddle PE, while CDT can be the method of choice in cases where there are more distal emboli, where aspiration might not be that useful. In select cases, a complementary approach, with aspiration thrombectomy as an initial approach followed by CDT for residual thrombi, might also be useful.

### 7.3. Early Reperfusion Treatment in Unique or High-Risk Circumstances

In select intermediate-risk PE cases—particularly in the context of threatening anatomical features such as a saddle embolus, floating thrombus, or clot-in-transit—early thrombolysis (systemic or catheter-directed) might be considered despite the patients being normotensive. Although guidelines generally recommend anticoagulation alone for intermediate-risk PE, they support escalation to reperfusion in scenarios of impending hemodynamic decompensation, significant clot burden, or mobile intracardiac thrombi. Specifically, systemic thrombolysis may be justified when there is a high risk of rapid deterioration and traditional anticoagulation is not permissible [152]. Moreover, when anticoagulation might be contraindicated due to high bleeding risk, CDI may offer a targeted approach which might reduce bleeding risk, along with obtaining a rapid improvement in RV dysfunction if present and lowering of pulmonary pressures [152]. For cases of thrombus-in-transit or sizable right heart clots, aspiration thrombectomy might be a good first-line therapeutic option. Guidelines also endorse CDI as a potential first-line approach when anticoagulation cannot be initiated, particularly when there is a fear of sudden hemodynamic collapse [152]. In such situations, a multidisciplinary team consultation—ideally via a Pulmonary Embolism Response Team (PERT)—should guide individualized decisions (further discussed).

### 7.4. Inferior Vena Cava (IVC) Filter

In specific clinical scenarios, the placement of an IVC filter may be an essential therapeutic alternative for patients with PE. IVC filters are mechanical devices implanted into the IVC to intercept emboli originating from DVT, thereby preventing them from reaching the pulmonary arteries. While anticoagulation remains the first-line treatment for VTE, IVC filters are indicated when anticoagulation is absolutely contraindicated—such as in the presence of active bleeding, or when PE occurs or propagates despite adequate anticoagulation [153]. Contemporary IVC filters fall into two categories: permanent or optional (retrievable, convertible), with modern practice favoring retrievable types to mitigate long-term complications [154]. Clinical guidelines, including those from the Society of Interventional Radiology, generally advise against routine IVC filter placement in patients successfully managed with anticoagulants; instead, they reserve it for high-risk scenarios and emphasize the importance of timely filter retrieval as soon as it becomes safe to resume anticoagulation [154]. Recent studies further highlight that in high- and intermediate-risk acute PE cases, particularly when thrombolytic therapy is employed, IVC filters may enhance in-hospital survival, though their overall benefit remains debated and should be weighed against risks such as filter-related complications and the potential for increased DVT [155]. IVC filters should be reserved for select patients who cannot receive anticoagulation or who experience recurrent PE under therapy, with best outcomes achieved through judicious use and prompt retrieval.

## 8. Patient Outcomes

Analysis performed by Becattini et al. [2] evaluated patient outcomes according to the ESC risk stratification scheme [1]. It was found that among the entire cohort of 906 patients with PE, the 30-day all-cause mortality and PE-related mortality were 7.2% and 4.1%, respectively. Death among the intermediate–high-risk PE patients was 7.7%, 6.0% in intermediate–low-risk PE, and 0.5% in the low-risk PE population, highlighting room for improvement in regard to outcomes of intermediate-risk PE patients.

Grifoni et al. [12] described 209 patients with PE, of whom 65 were normotensive with signs of RV dysfunction. Of these, 10% (N = 6) developed shock and required escalation therapy consisting of thrombolysis. Half of them (N = 3) had relative/absolute contraindication to thrombolysis and died soon after admission. Those who were managed with thrombolysis (N = 3) were discharged a few days after admission.

In the PEITHO trial [122], 2.6% of intermediate–high-risk patients receiving tenecteplase and 5.6% of the placebo group patients demonstrated hemodynamic decompensation or died within 7 days after randomization. Mortality at 30 days was 2.4% in the tenecteplase group, as compared to 3.2% in the placebo group (*p* = 0.42).

## 9. Future Directions

Stratification and therapy for patients with PE and especially intermediate-risk PE is gaining growing interest. Internists, pulmonologists, cardiologists, radiologists, and emergency care specialists are all involved in the diagnosis, stratification, management, and treatment of these patients.

CTA remains the gold standard non-invasive diagnostic imaging modality due to its high spatial resolution and vascular contrast enhancement. However, interpretation of CTA can be time-consuming and prone to inter-observer variability, especially in busy emergency settings or in subtle cases. Artificial intelligence (AI), particularly deep learning (DL) models, has shown promise in automating and enhancing the diagnostic process. The current AI systems utilized in CT scans and MRI interpretations are starting to gain popularity in the radiology world. The convolutional neural network (CNN)-based deep learning approach has notably been the most popular AI tool. Several studies have investigated the diagnostic accuracy of PE detection and the persistent issue of inter-observer variability. For instance, it has been reported that among radiologists with at least two years of experience, four out of five failed to identify peripheral emboli in 4–6 of 290 reviewed patients [156]. The rate of missed incidental PEs varies but has been documented to reach as high as 44.8%. However, with AI assistance, this miss rate can be reduced to as low as 2.6% [157]. Retrospective analyses have revealed that AI was able to identify PE in up to 38% of cases initially missed by radiologists [158]. In one comparative study, AI demonstrated a lower positive predictive value than clinical reports (86.8% vs. 97.3%, *p* = 0.03) and slightly lower specificity (99.8% vs. 100%, *p* = 0.045), while maintaining comparable sensitivity and negative predictive value in detecting incidental PE [159].

A study conducted by Cheikh et al. [160] further supports the utility of AI in PE diagnosis. Their AI-based software flagged 219 suspected PE cases, 176 of which were confirmed. Notably, 19 of these were initially overlooked by radiologists. The AI system achieved higher sensitivity (92.6%) and negative predictive value (98.6%) compared to radiologists (90% and 98.1%, respectively). However, radiologists outperformed AI in terms of specificity (99.1% vs. 95.8%) and positive predictive value (95% vs. 80.4%). These findings underscore the complementary role of AI in enhancing PE detection, particularly by reducing false negatives, while radiologists maintain strengths in minimizing false positives. AI holds considerable promise in equaling or surpassing radiologists in PE detection. However, its widespread implementation depends on further validation for safety, reliability, and bias mitigation. Several AI tools for PE triage on CTA for PE have received FDA approval, yet their integration into clinical workflows still requires human oversight.

Currently, MRI/MRA has no defined role in both diagnosis and prognosis of PE patients. While MRI is non-inferior to CTA in regards to imaging and diagnosis of central PE (at the level of the lobar arteries), its resolution is inferior for demonstrating segmental and sub segmental emboli [161]. However, MRI can play a role in assessment of RV dysfunction, estimating elevated mPAP as well as allowing pulmonary perfusion imaging. Some use MRI when there is contraindication to CTA (iodine anaphylactic reaction, for example, severe renal failure). Future trials using this technique will guide its role in managing PE patients.

Several studies have focused their attention on new technology to assess RV function. 3-D echocardiography, speckle tracking, and contrast echo [162] are only some of these tools that can assess RV function with higher resolution of both global and segmental regional wall deformations and aid in the prognostic assessment of the intermediate-risk PE patients and the extent of its effect on RV function. The PESI/sPESI score is currently the most validated clinical scoring tool for assessing and determining patients’ clinical risk and there is limited data about the BOVA score [163], which performed well in stratifying normotensive PE patients into several risk groups and predicting 30-day complications among these patients. The search for the optimal scoring system is ongoing. However, even a sub-analysis of the PEITHO trial failed to adequately identify a score which will aid in prediction of which intermediate-risk PE patients might benefit the most from a more invasive approach consisting of thrombolysis [31].

Better identification and stratification of PE patients who will benefit from an initially more aggressive therapy is still problematic. The concept of PERT is evolving [164] and becoming more widespread, aiding in determining all reperfusion options available to these complex patients and tailoring the best possible treatment. PERTs are multidisciplinary teams often including cardiology, pulmonary/critical care, emergency medicine, hematology, interventional radiology, surgery, and pharmacy advisors that coordinate the assessment and treatment of patients with intermediate- or high-risk PE. PERTs were established to optimize complex decision-making and aid in facilitating expedited diagnosis and initiation of therapy. Observational studies have revealed that PERT implementation can meaningfully reduce time to diagnosis and initiation of therapy, shorten hospital stays, and in several cases, decrease patient mortality. One tertiary center observed a reduction in six-month mortality from 24% to 14% (a 43% relative risk reduction), alongside a decrease in length of stay from 9.1 to 6.5 days [165]. Another retrospective study demonstrated that direct PERT consultation, not merely the presence of a PERT, was associated with a lower 30-day mortality (5% vs. 20%), a 5.4-day shorter hospital stay, faster time to therapeutic anticoagulation, and reduction in in-hospital bleeding [166]. Though results vary across institutions, the growing evidence supports PERTs as a vital component of modern PE management, enabling timely, expert-guided intervention tailored to individual patient risk profiles.

## 10. Summary

Intermediate-risk PE patients are a “complex” group of patients. Currently there is a paucity of data regarding adequate stratification and optimal management of these patients, which leads to current recommendations of close monitoring and providing escalation therapy when signs of hemodynamic instability appear. While effective, the main limitation of administrating systemic thrombolysis for these patients is the issue of safety. Future research to establish markers and criteria identifying the subgroup of patients which has the highest risk for clinical deterioration is necessary. Whether systemic thrombolysis or a novel approach such as catheter-directed therapy will be the treatment of choice for this group of patients will probably be determined in the upcoming years.

## Figures and Tables

**Table 1:** Validation of PESI and sPESI in PE patients.

Study	Population	Outcome
Aujesky et al. (2005) [19]	367 PE patients divided into 5 risk classes according to PESI score	Low-risk patients (Class I/II) had ≤1% 30 days mortality risk while class V had 24% mortality risk
Chan et al. (2010) [22]	302 PE patients assessed for 30- and 90-day mortality according to the PESI score	No mortality was observed among class I-III risk groups. 30-day and 90-day mortality among the class IV group was 9.2% and 10.5%, respectively.
Dentali et al. (2013) [23]	538 PE patients assessed for long-term mortality according to the PESI score	12-month mortality risk was <5% among class I patients and 72% among class V patients. The PESI score can be a useful tool for assessing long-term mortality among PE patients

**Table 3:** Computed tomography (CT) findings suggestive of RV involvement in intermediate-risk PE.

Study	CT Finding	Main Findings	Comments
RV/LV RATIO > 0.9 [55] N = 13,162 Figure 2A	RV/LV ratio measured in transverse and 4-chamber view	Association with a 2.5-fold risk of all-cause mortality and a 5-fold risk of PE-related mortality	Validated in assessing RV dysfunction and mortality [56,62],
IVC Contrast Reflux [59] N = 365 Figure 2B	Reflux of contrast to IVC and hepatic veins (different degrees of reflux depending on vein involvement)	IVC reflux predicts 30-day mortality [59]	Most trials support its prognostic role in acute PE [59,67].
Decreased left atrial size [65] N = 756 Figure 2C	Left atrium is measured in CT of the pulmonary arteries	Decreased left atrial size (<62 mL) is associated with higher clot load in pulmonary arteries and higher mortality rates [65].	New technology is emerging evaluating left atrial dimensions and correlation with patient outcome
Mastora et al. (2003) [57] N = 36	Percentage of obstructed central and peripheral pulmonary vessel.	A higher Mastora score was associated with RV dysfunction and higher pulmonary artery pressure and enables quantitative assessment of acute PE based on CT findings.	Mastora score correlation with mortality or short-term prognosis is debatable [59,60]
Qanadli et al. (2001) [61] N = 54	Quantification and degree of obstructed vessel based on anatomic location	High correlation between Qanadli score and pulmonary angiography findings. Higher scores were correlated with RV dilation	Conflicting results regarding prognostic significance of the Qanadli score [60,62,63]
Right atrial (RA)/Right ventricle (RV) ratio (2015) [66] N = 79	Measured in the 4-chamber view. Pathological cut-off was-1.01	RA/RV diameter ratio was found to correlate with 30-day mortality among normotensive PE patients	More research is needed to validate this parameter.
Hassan et al. (2023) [68] N = 703	Association between CT findings and persistent hypoxemia in intermediate-risk patients	Small arterial vessel fraction and PA/Aorta diameter ratio were associated with higher risk of persistent hypoxemia at discharge in intermediate-risk PE patients	Retrospective single-center study

**Table 4:** (**A**) Troponin for the evaluation and stratification of patients with PE. (**B**) Cardiac biomarkers other than troponin used for evaluation and stratification of patients with PE.

(**A**)				
**Study**		**Participants**	**Outcomes**	**Comments**
Bajaj et al. (2015) [73]		Large meta-analysis of 26 trials.	All-cause mortality was higher in the positive troponin group (10.5% vs. 3.1%). PE-related mortality was higher in the troponin-positive group (OR-3.8, CI 2.74–5.27) Serious adverse events (composite of death, need for thrombolytics, endotracheal intubation, catecholamine infusion for sustained hypotension, cardiopulmonary resuscitation, or recurrent PE)	Elevated mortality in the positive troponin group regardless of type (T/I)
Daquarti et al. (2016) [74]		40 Patients with PE Median PESI-81	30% of patients had RV dysfunction which was associated with higher troponin levels (33.5 ng/L vs. 16 ng/L, *p* = 0.03)	Troponin levels may be associated with RV dysfunction
Kaeberich et al. (2015) [75]		682 normotensive PE patients	Objective was adjusting troponin levels to age. Outcome: 30-day adverse events Age < 75—troponin cut off-14 pg/mL Age > 75—troponin cut off-45 pg/ml	Troponin is a useful biomarker predicting adverse events and may be adjusted to age.
Keller et al. (2015) [76]		129 normotensive PE patients	Troponin was associated with RV dysfunction (OR 3.95, CI 1.95–8.02, *p* = 0.00014)	Troponin is correlated with submassive PE. NPV-73%
Hakemi et al. (2015) [77]		298 patients with PE	Patients with a negative high sensitive troponin had better survival rates irrespective of clinical risk	Negative HS-troponin may serve as a tool for identifying low-risk patients
Becattini et al. (2007) [78]		Meta-analysis of 20 trials	Elevated troponin was associated with both worse short-term mortality and adverse outcomes	Elevated troponin is associated with high mortality among hemodynamically stable patients
Stein et al. (2010) [79]		1273 hemodynamically stable PE patients	Increased troponin+ RV enlargement had higher mortality rate compared to negative troponin and normal RV (10.2% vs. 1.9%)	Positive troponin + RV enlargement are strong predictors of adverse outcome among normotensive PE patients
Meyer et al. (2000) [80]		36 PE patients	62% patients with RV dilation had positive troponin.	Positive troponin also correlated with more segmental defects in V/Q scans
Pruczczyk et al. (2003) [81]		64 normotensive PE patients	Repetitive measurements of cardiac troponin At a 6 h interval. The positive troponin group was at high risk for a complicated course (PE- related death)	Repetitive measurements of elevated cardiac troponin are important for risk stratification in normotensive PE patients
(**B**)				
**Biomarker**	**Study**	**Participants**	**Outcome**	**Comments**
Brain natriuretic peptide (BNP)	Lega et al. (2009) [82]	23 studies—1127 patients	Elevated natriuretic peptide is associated with all-cause mortality (OR-6.2) and PE-related mortality (OR-5.0)	Natriuretic peptides can be used as risk stratification among PE patients
	Wolde et al. (2003) [83]	110 PE patients	BNP cut-off of 21.7 pmol/L predicts an NPV of 99% for an uneventful outcome	
	Pieralli et al. (2006) [84]	61 normotensive PE patients	57% had evidence of RV dysfunction. A BNP level of <85 pg/mL excluded RV dysfunction. Higher levels were associated with RV dysfunction	BNP was a powerful predictor of adverse outcomes with increased levels associated with RV dysfunction
	Kucher et al. (2003) [85]	73 patients with acute PE	BNP levels of <50 pg/mL predicted a benign clinical course	Low levels of BNP may be considered to identify low-risk patients
	Lankeit et al. (2014) [86]	688 normotensive PE patients	NT pro BNP cut-off levels of 600 pg/mL were associated with adverse outcomes.	Elevated levels of NT pro BNP are associated with mortality or clinical deterioration. Low levels (<600 pg/mL) are not sufficient to define low risk
Heart-type fatty acid-binding protein (H-FABP)	Ruan et al. (2014) [87]	Meta-analysis of 6 trials—618 patients	Elevated H-FABP was associated with 30-day mortality (OR-40). Sensitivity and specificity for death and SAE—98% and 86%, respectively	H-FABP is a useful prognostic factor among PE patients
	Liu et al. (2015) [88]	Meta-analysis of 6 studies, 594 patients	Elevated H-FABP was associated with elevated risk of death, cardiopulmonary resuscitation, endotracheal intubation, use of vasopressors, thrombolysis, surgical embolectomy, admission to the intensive care unit, or mortality in patients with acute PE.	H-FABP is a predictor of adverse events among PE patients
	Bajaj et al. (2015) [89]	Meta-analysis of 11 studies—1680 patients (9 studies with hemodynamically stable patients)	H-FABP was associated with complicated course-death, need for thrombolytics, endotracheal intubation, catecholamine infusion for sustained hypotension, CPR, or recurrent PE, as well as 30-day PE-related mortality and RV dysfunction	Prognostic sensitivity and specificity of H-FABP were 90% and 70%, respectively. In predicting 30-day mortality
	Dellas et al. (2010) [90]	126 normotensive PE patients	Higher H-FABP among patients with short-term complications: 30-day mortality, need for catecholamine use and intubation, as well as long-term mortality	H-FABP can predict both short- and long-term mortality among normotensive PE patients
	Boscheri et al. (2010) [91]	101 patients with intermediate-risk PE (RV dysfunction without signs of shock)	H-FABP was a predictor of mortality	Useful and novel biomarker among intermediate-risk PE
Lactate	Vanni et al. (2013) [92]	270 PE patients	Higher plasma lactate was associated with higher mortality as well as a composite of mortality, progression to shock, mechanical ventilation, and CPR.	Lactate elevation is associated with tissue hypoperfusion and can be seen prior to clinical hemodynamic instability.
	Vanni et al. (2017) [93]	994 normotensive PE patients	Adding plasma lactate to the clinical BOVA score identified more patients who are prone to deterioration (hemodynamic collapse and death within 7 days of diagnosis)	Plasma lactate may be used for risk stratification among normotensive PE patients
D-Dimer	Becattini et al. (2012) [94]	Meta-analysis of 22 studies including both stable and unstable PE patients	Higher D-dimer levels associated with both short- (30-day) and long-term (3-month) mortality. Conflicting results regarding correlation with RV dysfunction or thrombotic burden	Prognostic value of D-dimer could not be obtained. Because of low specificity, D dimer alone cannot be used for risk stratification and decision-making algorithm among intermediate-risk PE patients
Copeptin	Hellenkamp et al. (2015) [95]	Prospective single-center study including 268 normotensive PE patients	Elevated copeptin levels were associated with a 5.4-fold increased risk of adverse 30-day outcome	Copeptin might be helpful for risk stratification of normotensive patients with PE, especially if integrated into a biomarker-based algorithm.
	Hellenkamp et al. (2018) [96]	European multicenter study that validated the prognostic impact of copeptin in 843 normotensive patients with acute PE	Patients with copeptin ≥24 pmol·L had a 6.3-fold increased risk for an adverse outcome and a 7.6-fold increased risk for PE-related death	Supporting the concept that copeptin provides information on the hemodynamic impairment due to acute RV failure

**Table 5:** Main studies evaluating thrombolysis/catheter-guided therapy vs. anticoagulation in intermediate-risk/submassive PE.

**Thrombolytic Therapy**				
**Study**	**Participants**	**Objective**	**Outcome**	**Conclusion**
Gao et al. (2015) [121] Meta-analysis of RCT	Total of 8 studies, 1755 patients Meta-analysis, RCT	Thrombolytic therapy vs. anticoagulation in intermediate-risk PE	Lower mortality in thrombolytic group (RR 0.52, 95% CI (0.28–0.97) Higher major bleeding in thrombolytic patients (RR 3.35, 95% CI, 2.03–5.54)	Intermediate-risk patient may derive benefit from thrombolytic treatment. Bleeding risk must be taken into consideration.
Konstantinides et al. (2002) [124] Prospective RCT	118-heparin + alteplase. 138-heparin + placebo Prospective, RCT	Thrombolytic therapy vs. anticoagulation in submassive PE	No mortality benefit (3.4% thrombolytic vs. 2.2% heparin, *p* = 0.71). Less clinical deterioration in thrombolytic group (24.6% vs. 19.2%, *p* = 0.004)	Thrombolytic therapy can prevent clinical deterioration
Fasullo et al. (2011) [125] Prospective RCT	37-thrombolysis 35-heparin Prospective, RCT	Thrombolytic effect vs. heparin on clinical and echocardiographic parameters within 180 days in submassive PE	Thrombolytic group had significant early improvement in RV function which was sustained after 180 days	Thrombolytic therapy in submassive PE improve RV function both in the short and long term
Sharifi et al-MOPETT trial (2013) [128] Prospective RCT	121 PE patients with “moderate PE” involvement of 2 lobar or right/left main pulmonary arteries	Test “safe dose” thrombolytic therapy—≤50% TPA regular dose. Primary outcomes of pulmonary hypertension and composite of pulmonary hypertension and recurrent PE	Pulmonary hypertension and combined outcomes developed in 57% of control group and 16% of “safe dose” group (*p* < 0.001) No significant difference in mortality, recurrent PE, or bleeding	“Safe dose” thrombolysis can be considered as safe and effective at reducing pulmonary hypertension among stable PE patients
PEITHO (2014) [122] Prospective RCT	506-tenecteplase 499-placebo Prospective, RCT	Thrombolytic therapy vs. heparin in intermediate-risk PE	Death or hemodynamic decompensation was lower in thrombolytic therapy compared to placebo (2.6% vs. 5.6%, OR 0.44, 95% CI, 0.23–0.87, *p* = 0.02).	Thrombolytic therapy prevented hemodynamic decompensation but increased the risk of major hemorrhage and stroke
Nakamura et al. (2014) [129] Meta-analysis of RCT	Total of 6 studies, 1510 patients. Meta-analysis. Only RCTs were included	Thrombolytic therapy vs. Heparin in submassive PE	No difference in combined outcome of mortality and recurrent PE (3.1% vs. 5.4%, RR 0.64, *p* = 0.2). Significant reduction in combined all-cause death and clinical deterioration (3.9% vs. 9.4%, RR 0.44, *p* < 0.001). No significant major bleeding in thrombolytic group.	adjuvant thrombolytic therapy prevents clinical deterioration
Kline et al. (2014) [120] Prospective RCT	40-Tenecteplase 43-Placebo Prospective, RCT	Thrombolysis vs. heparin in submassive PE	Adverse outcome (death, circulatory shock, major bleeding, recurrent PE, poor functional capacity) was higher in the placebo group at 90 days (37% vs. 15%, *p* = 0.017)	Better outcome in thrombolytic group
Xu et al. (2015) [126] Meta-analysis of RCT	Total of 7 trials, 1631 patients Meta-analysis Only RCTs were included	Efficacy and safety of thrombolysis in intermediate-risk PE	Trend to reduction in all-cause mortality (OR 0.6, CI 0.34–1.06, *p* = 0.08), recurrent PE (OR 0.34, CI 0.15–0.77, *p* = −0.01), and clinical deterioration (OR 0.27, CI 0.18–0.41, *p* < 0.01) with higher minor bleeding in thrombolytic group. No difference in major bleeding	Thrombolysis is a viable option in intermediate-risk PE, associated with reduction in PE recurrence. Clinical deterioration without higher major bleeding
Chatterjee et al. (2014) [130] Meta-analysis of RCT	Total of 8 trials, 1775 patient. Meta-analysis. Only RCTs were included	Thrombolytic therapy vs. anticoagulation in intermediate-risk PE	Thrombolysis was associated with lower all-cause mortality (OR 0.53, CI 0.32–0.88. NNT = 59. Thrombolysis was associated with higher risk of major bleeding (OR 2.73, CI 1.91–3.91). NNH = 18	Thrombolysis was associated with lower mortality and increased risk of major bleeding and intracranial hemorrhage.
**Catheter-Directed Thrombolysis (CDT)**				
**TRIAL**	**Participants**	**Objective**	**Outcome**	**Conclusion**
ULTIMA (2014) [131] Prospective RCT	59 patients (30 treated with CDT, 29 with AC)	Ultrasound-assisted catheter-directed thrombolysis vs. anticoagulation	RV/LV ratio was significantly reduced in USA group vs. anti coagulation (0.3 ± 0.2 vs. 0.03 ± 0.16, *p* < 0.001)	USAT was superior to anti coagulation in reversing RV dilation at 24 h
CANARY (2022) [132] Prospective RCT	94 patients Open-label, RCT Stopped prematurely d/t COVID-19	CDT vs. anti coagulation	3-month RV/LV ratio was significantly lower with the CDT (0.7 (0.6–0.7) vs. 0.8 (0.7–0.9), *p* = 0.01) CDT patients experienced lower rate if composite of death or RV/LV > 0.9	Hypothesis generating for improvement in efficacy outcome with CDT. Trial was terminated prematurely.
SUNSET sPE (2021) [133] Prospective RCT	81 patients with submassive PE. 1:1 randomization to ultrasound-assisted thrombolysis vs. standard catheter-directed thrombolysis	1:1 randomization to ultrasound-assisted thrombolysis vs. standard catheter-directed thrombolysis	48 hours’ thrombus burden was reduced in both groups. No significant difference was seen between the groups.	No significant difference in thrombus clearance between ultrasound-assisted and standard catheter-directed thrombolysis
SEATTLE II (2015) [134] Prospective single-arm	119 patients Single-arm, multicenter	Efficacy and safety of USAT using EKOS system	Mean RV/LV diameter ratio decreased from baseline to 48 h (1.55 vs. 1.13, *p* < 0.001). Mean PA pressure decreased from 51.4 to 36.9, (*p* < 0.001)	USAT decreases RV dilation and reduces pulmonary hypertension as well as anatomic thrombus burden
OPTALYSE PE (2018) [135] Prospective, parallel group	101 patients Prospective, multicenter, parallel-group	USAT vs. 1 of 4 USAT thrombolytic regimens	Improvement in RV/LV diameter ratio was seen in all subgroups	USAT with low-dose thrombolysis was associated with improved RV function and reduced clot burden
KNOCOUT PE (2024) [136] Prospective, single-arm	489 patients Prospective Multicenter Single-arm	Safety and efficacy of ultrasound-facilitated catheter-directed thrombolysis	Major bleeding within 72 h in 1.6% of patients. All-cause mortality at 30 days—1.0%. QoL improvement in 41.1%	Low mortality and bleeding using catheter-directed thrombolysis
Pasha et al. (2022) [137] Meta-analysis	11,932 patients 8 observational studies	Safety and efficacy of systemic and catheter-directed thrombolysis	CDT was associated with lower in-hospital mortality (RR 0.52, 95%CI (0.4–0.68)). ICH was lower in CDT group (RR 0.66, 95%CI (0.47–0.94))	Non randomized trial suggests better efficacy and safety of CDT compared to systemic thrombolysis
**Mechanical Thrombectomy**				
**TRIAL**	**Participants (Intermediate-Risk PE)**	**Objective**	**Outcome**	**Conclusion**
FLARE (2019) [138] Prospective, single-arm	104 patients Single-arm study	Safety and effectiveness of FlowTriever System (Inari) in intermediate–high-risk PE patients	RV/LV ratio reduction was 0.38 (25.1%, *p* < 0.0001). Major bleeding—1%	Mechanical thrombectomy with FlowTriever was safe and effective in intermediate–high-risk patients
FLASH (2023) [139] Prospective, single-arm	799 patients in US cohort. Single-arm study. 76% of patients had intermediate–high-risk PE	Safety and effectiveness of mechanical thrombectomy using FlowTriever	23% reduction in mean pulmonary artery pressure (*p* < 0.001). 63% of patients had no overnight intensive care unit stay. RV/LV ratio decreased from 1.23 ± 0.36 to 0.98 ± 0.31 (*p* < 0.001)	Mechanical thrombectomy with the FlowTriever system had a favorable safety profile and improvement in hemodynamic and functional outcomes
PEERLESS (2025) [140] Prospective, RCT	550 patients Prospective multicenter RCT	Large bore mechanical thrombectomy (LBMT) vs. catheter-directed thrombolysis	Composite of all-cause mortality, intracranial hemorrhage, major bleeding, clinical deterioration and/or escalation to bailout and postprocedural ICY admission. Primary outcome was lower in LBMT compared with CDT (win ratio 5.91 (95%CI 3.68 = 6.97)	LBMT had lower rates of clinical deterioration and/or bailout and postprocedural ICU use compared with CDT
EXTRACT PE (2021) [141] Prospective, single-arm	119 patients. Intermediate–high-risk PE	Efficacy and safety of thrombus aspiration with the Indigo aspiration system	RV-to-LV ratio decreased on average by 0.43 ± 0.26 (95% CI 0.38–0.47, *p* < 0.0001), PAP pressure was significantly reduced. 2 patients had major bleeding. Overall, 1.7% major adverse event rate	
**Ongoing Trials**				
**Trial**	**Participants**	**Objective**	**Outcome**	
HI-PEITHO (2022) [142] RCT	Approximately 406 Intermediate–high-risk PE patients	USCDT vs. anticoagulation	Primary: PE-related mortality, PE recurrence, and hemodynamic decompensation. Secondary: changes in LV/RV ratio, cardiorespiratory support, GUSTO bleeding	
STRATIFY (2024) [143] RCT	210 intermediate–high-risk PE	(1) unfractionated heparin (UFH)/low-molecular-weight heparin (LMWH), (2) UFH/LMWH + 20 mg rtPA/6 h intravenously (IV), or (3) UFH + 20 mg rtPA/6 h via USAT.	Primary: Reduction in clot burden. Secondary: bleeding complications, duration of index admission, FIO2, blood pressure, respiratory and heart rate at follow-up CT, mortality, incidence of tricuspid regurgitation, mortality, QoL and 6 min walk test at 3 months	
PE-TRACT (2025) [144] RCT	500 intermediate-risk PE	CDT + anticoagulation vs. anticoagulation	Primary: Peak Vo2 and NYHA class at 12 months	
STRIKE-PE (2025) [145] Prospective Observational	600 Intermediate- and high-risk PE	Indigo aspiration system	Change in RV/LV ratio, device-related death, major bleeding, device-related clinical deterioration, device-related pulmonary vascular injury, and device-related cardiac injury—at 48 h after procedure. Interim analysis (150 patients) shows 25.7% reduction in RV/LV ratio, rate of combined adverse effect—2.7%, serious adverse effects—1.3%, 30-days all-cause mortality—2.0% [146]	
PEITHO 3 (2022) [147]	Planned 650 patients with Intermediate–High-risk PE	Reduced-dose Alteplase vs. placebo with heparin	Composite of all-cause death, hemodynamic decompensation, or objectively confirmed recurrent PE within 30 days of randomization	

## Data Availability

The original contributions presented in this study are included in the Article/Supplementary Material. Further inquiries can be directed to the corresponding author.

## Supplementary Materials

The following supporting information can be downloaded at: https://www.mdpi.com/article/10.3390/jcm14176215/s1, Table S1: PESI and sPESI scores as presented in the ESC guidelines on the diagnosis and management of pulmonary embolism [19].
