# Supplementary material for: Intermediate-Risk Pulmonary Embolism: Patients’ Stratification, Prognosis, and Therapeutic Options—Time to Pay Attention to the Middle Child
Source: J Clin Med. 2025 Sep 3;14(17):6215. doi: 10.3390/jcm14176215 (PMC12429051; doi:10.3390/jcm14176215)
Supplement: Supplementary file 1 [file jcm-14-06215-s001.zip › jcm-3795676-supplementary.pdf]

Supplementary tables:

Table S1: PESI and sPESI scores as presented in the ESC guidelines on the diagnosis and management of pulmonary embolism[19]

**Parameter**	**PESI**	**sPESI**
**Age**	Age in years	1 point-Age>80
**Male sex**	+10	
**Cancer**	+30	1 point
**Chronic heart failure**	+10	
**Chronic pulmonary disease**	+10	1 point
**Pulse rate>110 b.p.m**	+20	1 point
**Systolic blood pressure<100 mmHg**	+30	1 point
**Respiratory rate>30 breath per minute**	+20	
**Temperature<36˚C**	+20	
**Altered mental status**	+60	
**Arterial oxyhemoglobin saturation<90%**	+20	1 point
	Class I:<65 points. (30-day mortality risk-0-1.6%)<br><br>Class II: 66-85 points (30-day mortality risk 1.7-3.5%)<br><br>Class III: 86-105 points (30-day mortality risk 3.2-7.1%)<br><br>Class IV: 106-125 points (30-day mortality risk 4.0-11.4%)<br><br>Class V: >125 points (30-day mortality risk 10.0-24.5%)	0 points- 30-day mortality risk 0-2.1%<br><br>≥ 1 points: 30-day mortality risk 8.5%-13.2%

***Adapted from** D. Aujesky *et al.*, “Derivation and validation of a prognostic model for pulmonary embolism,” *Am. J. Respir. Crit. Care Med.*, vol. 172, no. 8, pp. 1041–1046, Oct. 2005.
